# Specimen of Reporting

**Published:** 1831-01-01

**Authors:** 


### EDINBURGH SURGICAL HOSPITAL.

FISTULA IN ANO.

*Report.*

"James Trainer, æt. 38, from Leith, recommended by Dr. Kirk, was admitted on the 25th of June. On the left side of the anus there is an induration of the integuments and subjacent parts, in the centre of which there is an opening which discharges matter. A probe introduced at this aperture passes up a winding sinus, which opens into the gut about an inch from the verge of the anus, at a different part of the circumference. He complains of pain, which prevents him from sitting, and which is particularly distressing when he goes to stool.

Two months ago, he experienced a difficulty in evacuating the rectum, and felt as if there was a lump on the left side of the gut, which occasioned great pain when it was subjected to pressure. Poultices were applied for a fortnight; matter was discharged into the gut, and the pain abated. A few days afterwards, he felt an external swelling, which was poulticed and opened by Dr. Kirk, who, recognizing the nature of the case, advised him to repair to the Hospital, where he could have more attention paid to him than at home.

I divided the septum on the 26th, and he was dismissed cured on the 10th of July."*

FISTULA IN ANO.

*Analysis.*

James Trainer; two months ago had an abscess on the side of the rectum, which remained fistulous, and communicated with the gut by a winding sinus, an inch above the verge of the rectum. The septum was divided, and the patient discharged cured in a few days.

*Remarks.* Why Dr. Kirk should have sent the above patient to the hospital, or why Mr. Syme should have deemed it necessary to publish it to the world in the 19th century, we cannot even conjecture! We defy any person to point out the minutest iota of interest or utility in the case, or to shew any one *thing* (unless, as Mirabeau asserted, "words are things") in the report, which is not contained in the four or five lines of analysis. We grant, indeed, that neither the report nor the analysis is worth the fiftieth part of the paper on which they are spread. The *illustration* of the manner and matter of some hospital reports is the only useful part of the business, because it will prove a hint to reporters which will not be thrown away. But we must give one more example.

* Edinb. Journal, Oct. 1, 1830.

